# Accelerating Resistance Breeding: Emerging Methods to Identify and Validate Plant Immunity Genes

**DOI:** 10.3390/plants15050685

**Published:** 2026-02-25

**Authors:** Ziyu Liu, Klaas Cloots, Koen Geuten

**Affiliations:** 1 Unit of Molecular Biotechnology of Plants and Microorganisms, Department of Biology, KU Leuven, Kasteelpark Arenberg 31, 3001 Leuven, Belgium; ziyu.liu@kuleuven.be (Z.L.); klaas.cloots@kuleuven.be (K.C.); 2 KU Leuven Plant Institute (LPI), KU Leuven, Kasteelpark Arenberg 31, 3001 Leuven, Belgium

**Keywords:** plant immunity genes, GWAS, NLR, omics, plant-pathogen interactions, CRISPR, protoplasts, resistance breeding

## Abstract

Plant pathogens are a major cause of crop yield loss, making disease resistance breeding crucial for crop improvement. Plants have evolved innate immune systems, mediated by immune-related genes such as nucleotide-binding site leucine-rich repeat (NLR), pattern-recognition receptors (PRR) and susceptibility genes, which are essential resources for breeding disease-resistant plants. To identify immunity genes, extensive genetic approaches that examine the association between resistance phenotypes and genomic regions have been applied with great success. While genetic methods remain important for identifying immunity genes, novel strategies that rely on functional rather than genetic association with disease resistance offer unique advantages. For example, mutagenesis with *R* gene enrichment sequencing (MutRenSeq) enabled the identification of wheat resistance genes *Sr22* and *Sr45* by comparing the NLRomes of resistant and susceptible lines while single-cell RNA sequencing resolved cell-type-specific responses to pathogen infection and revealed *ZmChit7*, especially in maize epidermal and guard cells. These approaches reach beyond existing natural variation, can accelerate experimental timelines, reduce the experimental scale, and provide mechanistic insights into pathogen resistance. This review discusses emerging techniques that generate focused candidate immunity gene lists or accelerate their validation, as both are required to identify causal variants for resistance breeding. We consider advances in RenSeq-derived methods, spatial omics, proximity labelling, computational prediction, Clustered regularly interspaced short palindromic repeats (CRISPR) screens, and cell death assays. These approaches are reshaping resistance breeding pipelines beyond association-based discovery. By discussing the strengths and limitations of these emerging methods and their combinations, we outline current opportunities and future directions to help plant pathologists to more effectively identify and validate plant immunity genes.

## 1. Introduction

About 17–30% of major food crop yields are lost globally due to plant diseases and pests [1]. Plant pathogens not only cause direct loss of yield (food availability), but also impair food security and nutrition (food utilization efficiency), for example, through toxin accumulation [2]. On the other hand, the growing population and climate change impose additional demands on crop productivity [3,4]. To keep up with pathogen adaptation and the changing patterns of pathogen spread, developing disease-resistant crops is crucial. This requires the identification of key immunity genes and a thorough understanding of their function. The plant innate immune system primarily consists of pattern-triggered immunity (PTI) and effector-triggered immunity (ETI), which mainly recognize pathogen signals at the cell surface and within the cell, respectively [5,6]. Upon recognition of pathogens, these systems activate a cascade of downstream immune responses, such as calcium influx, reactive oxygen species burst, and hormonal signaling [7,8]. Meanwhile, a functionally variable class of plant genes, referred to as susceptibility genes (*S* genes), can facilitate infection by assisting pathogen penetration, proliferation, transmission, or suppression of host immunity. Knockout or suppression of *S* genes may effectively disrupt pathogen replication [9,10]. Developing methods to identify and characterize these immunity-related genes is therefore of great significance for the breeding of disease-resistant crops.

Historically, genetic approaches have made landmark contributions in the identification of plant immunity genes. Map-based cloning has identified key plant immune genes such as *Solanum lycopersicum* L. (tomato) *Pto*, *Hordeum vulgare* L. (barley) *Rpg5*, *Triticum aestivum* L. (wheat) *Sr33* and so on [11,12,13]. Quantitative trait locus (QTL) mapping applies controlled crosses and molecular markers to locate traits in the genome, establishing quantitative links between genomic regions and phenotypic traits [14], and is subsequently combined with map-based cloning to localize causal genes [15,16]. These methods have led to the identification of critical plant immune-related genes, especially nucleotide-binding site leucine-rich repeat (NLR) genes and pattern-recognition receptors (PRRs). Based on the breakthroughs in high-throughput genotyping (e.g., gene chips and next-generation sequencing), the high-quality reference genomes, and the innovation of statistical models, genome-wide association studies (GWAS) have been developed. GWAS explores the natural genetic variation within association panels and has been extensively applied to study pathogen resistance in a wide range of plant species [17], leading to the identification of a series of key plant immune genes as well as many QTLs [18,19,20]. The discovery that many candidate genes are not limited to traditional NLR or PRR genes has further challenged and expanded our perception of plant immunity [17]. Furthermore, with advances in technology and analytical methods, such as automated high-throughput phenotyping platforms [21], multi-locus GWAS [22], integration of multi-omics data [23,24,25], and the use of pan-genomes [26,27], GWAS experiments have been continuously improving.

Despite having been developed and applied for decades, genetic methods exhibit some inherent limitations. A primary consideration is that they are often time- and resource-intensive: linkage mapping, for instance, can require several years to localize causal genes [17]. Large-scale sequencing, phenotyping, and substantial experimental space are frequently beyond the capacity of many laboratories. Additionally, some immunity genes can be difficult to detect using genetic approaches, such as rare or minor effect loci, structural variants (for example, clustered or large-scale gene deletions), and non-sequence-based variations (including epigenetic and transcriptional regulatory changes). The resolution of genetic approaches also depends on the population size, population structure and recombination rate, which sometimes does not allow the validation of actual causal variants if the identified genomic region contains too many candidate genes [17,28]. Finally, they establish a relationship between sequence variation and phenotypic effect, but without an understanding of the causal mechanism [29,30], while this mechanism may implicate other relevant genes and factors involved in the disease.

**Figure 1:**
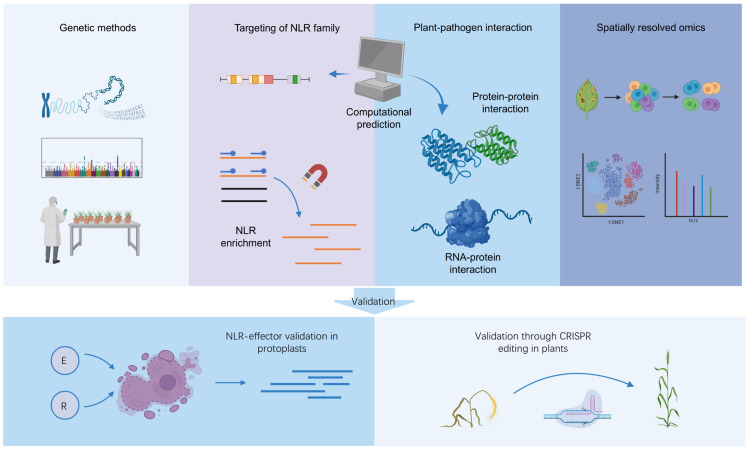
Overview of emerging approaches for identifying and validating candidate immunity genes supplementary to genetic approaches. Genetic methods have long been the primary tool for identifying genes involved in plant immunity. Emerging approaches that focus on NLR genes, plant-pathogen interactions, and multi-omics analyses provide supplementary information to genetic studies. In addition, new validation techniques using protoplast systems and high-throughput CRISPR editing can further accelerate the functional validation of immunity genes. E, effector. R, NLR genes. Created in BioRender. Liu, Z. (2025) https://BioRender.com/rox6hyi (accessed on 20 January 2026).

In recent years, remarkable progress has been made in methods that identify plant immunity genes based on functional instead of genetic association with disease resistance. For instance, resistance gene enrichment sequencing and its derived methods specifically target the NLR gene family, thereby offering higher resolution and efficiency compared to the broad genomic scope of GWAS [31,32,33]. Additionally, interactomics can provide evidence on the molecular level to explain possible immune mechanisms, whereas spatially resolved omics approaches such as single-cell RNA-sequencing enable the analysis of cell-specific changes during pathogen infection, which greatly improves the signal-to-noise ratio [34]. The power of these methods is further enhanced by recent computational tools that can improve the prediction of NLR genes and protein–protein interaction [35,36]. Here, we review these emerging, function-based methods that can supplement traditional genetic approaches in identifying candidate immunity genes (Table 1; Figure 1). The identification of potential immunity genes based on their functional correlation to disease resistance instead of their genetic association with available natural trait variation provides key advantages in: (1) accessibility by avoiding the need for large populations and fine mapping, (2) exploration of candidate genes that were previously hidden due to limited natural variation, (3) revealing underlying resistance mechanisms to guide research, and (4) facilitating breeding through CRISPR-based validation when natural variation is insufficient.

Functional validation of candidate immunity genes identified by any method is essential for their application in resistance breeding, as only then can the actual causal variants that explain disease resistance be identified [29]. However, a major bottleneck in validating the substantial candidate gene lists remains the scale of such validation experiments [37]. Therefore, we also discuss the recent progress in high-throughput methods for validating candidate immunity gene lists, including pooled CRISPR screening and protoplast validation approaches (Figure 1). To summarize the main advantages and limitations of all discussed methods, a supporting overview is presented in Table 1.

## 2. Alternative Approaches to Identify Candidate Immunity Genes

### 2.1. Targeted NLR Gene Screening

NLR genes play a pivotal role in plant immunity as core components of the effector-triggered immunity system. They recognize pathogen effectors, thereby activating robust immune responses, including hypersensitive cell death [5,6]. Compared with non-targeted genetic approaches, methods focusing on the NLR gene family are more immunity-related and reduce experimental and cloning work. When combined with functional validation pipelines such as MutRenSeq, subsequent laborious characterization is minimized. However, the NLR gene family is highly diverse due to strong pathogen-driven selection [38]. This diversity is striking in both NLR copy number variation across species and mutations within species, posing substantial challenges for identification. Functional characterization of plant NLRs remains largely incomplete, as even in *Arabidopsis thaliana*, only 17.6% of NLRs have been characterized. Moreover, reliance on a single reference genome severely underestimates NLR diversity. Pan-genome analyses show that many NLRs are absent from reference assemblies, for example, 60% in *Brassica oleracea* and 50% in *Brassica napus* are missing [39]. These unexplored NLRs represent vast untapped resources for disease resistance in plants.

#### 2.1.1. Revealing the NLRome: NLR Gene Annotation

The construction of a plant NLR pan-genome, whether based on whole-genome assemblies or RenSeq-derived contigs, presents a significant challenge for comprehensive NLR annotation [39,40]. The NLR gene family is characterized by a conserved NB-ARC domain (nucleotide-binding adaptor shared by APAF-1, certain *R* gene products, and CED-4), a C-terminal LRR (leucine-rich repeat) domain, and a highly variable N-terminal domain. The N-terminal domain is often classified into three types: TIR (Toll/interleukin-1 receptor), CC (Rx-type coiled-coil), or RPW8-type coiled-coil (Resistance to Powdery Mildew 8), which correspond to three subclasses of NLRs: TNLs, CNLs, and RNLs, respectively [41]. Beyond the NLR family, PRRs and LRR-like receptors are also widely considered in many annotation tools [35,42]. Multiple bioinformatics tools have been developed based on these sequence specificities.

Common NLR annotation typically relies on two approaches: motif alignment and domain-based detection. For example, NLR-Parser uses motif alignment to predict NLR genes genome-wide [43]. Building on this, NLR-Annotator defines an NLR locus as a genomic region containing one NB-ARC domain and possibly one or more LRR domains [44,45]. However, given NLR diversity, domain-based detection of complete functional domains rather than short conserved motifs has become the predominant strategy. DRAGO2, for instance, employs alignments form NCBI blast of known resistance genes to construct hidden Markov models for screening LRR, kinase, NB-ARC, and TIR domains [46]. Based on DRAGO2, the Plant Resistance Gene Database was established, containing experimentally validated and predicted NLR genes, including NLRs, PRRs, and LRR-like receptors [35]. Similarly, NLGenomeSweeper identifies NB-ARC domains first and then uses InterProScan to detect LRR domains within a 10 kb flanking region to confirm NLR candidates [47]. Other tools, such as NLRtracker, RGAugury, and R-predictor, rely on common domain detection programs such as InterProScan, nCoils, pfam_scan, and Phobius [48,49,50]. Notably, the RefPlantNLR database, comprising 481 experimentally validated plant NLRs, remains the most reliable resource, and the NLRtracker tool derived from it shows superior accuracy for CC domain annotation [49].

Recently, machine learning approaches have been applied to capture hidden sequence patterns missed by traditional methods. Some machine learning-based tools have been developed for NLR and motif prediction, including NLRexpress [51] and ESM-LRR [50], which employ multi-layer perceptrons and protein language models, respectively. By integrating multi-dimensional data such as amino acid frequency, physicochemical properties of NLR motifs (hydrophobicity scale, charge and volume), and the combination of NLR motifs, machine learning models can autonomously detect hidden features, enabling the identification of non-canonical resistance genes sharing functional similarities beyond typical domains or motifs [51].

However, current machine learning approaches rely on existing NLR genes in model plants. If the training data lacks examples of highly divergent LRR or NLR genes, models may fail to recognize them [51,52]. Although NLR gene prediction tools can annotate NLR genes genome-wide, they cannot associate NLR genes with specific pathogens or effectors. Therefore, generating a more accurate NLR gene annotation with these prediction tools mainly supports experimental NLR gene discovery pipelines, for example via RenSeq [31,40]. Another limitation is that most tools are optimized for their own datasets, raising concerns about their applicability across diverse plant species and genomic, transcriptomic, or proteomic resources.

#### 2.1.2. Targeted NLR Gene Identification: MutRenSeq & AgRenSeq

Resistance gene enrichment sequencing (RenSeq) was developed for targeted identification of members of the NLR gene family, in which NLRs are captured using biotinylated oligonucleotides designed from NB-LRR-like sequences and subsequently sequenced [31]. This method has greatly expanded and reannotated NLR repertoires in many crops [52]. When combined with single-molecule real-time (SMRT) sequencing to obtain full-length NLR sequences, SMRT-RenSeq enables discrimination of highly similar NLRs that are indiscernible in short reads [53,54]. Compared to QTL mapping or GWAS, RenSeq enriches NLRs to reduce genome complexity, making it applicable to wild species without annotated genomes and to crops with complex polyploid genomes by using probes designed from related species. Among RenSeq-derived methods, MutRenSeq and AgRenSeq are the most powerful for NLR identification.

**Figure 2:**
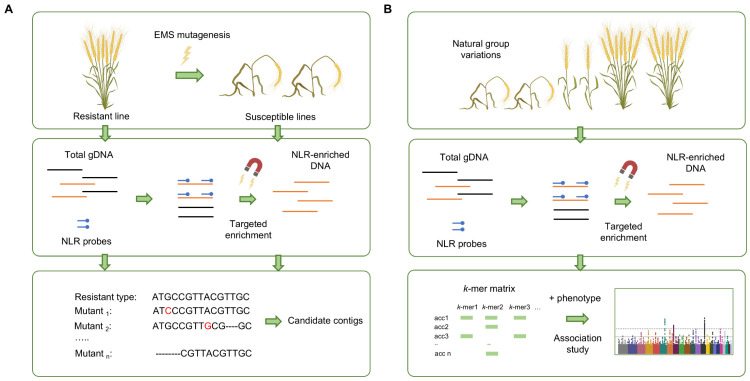
Schematic overview of the NLR enrichment methods MutRenSeq and AgRenSeq. (**A**) MutRenSeq employs EMS mutagenesis to generate loss-of-resistance mutants from a resistant line, followed by NLR enrichment and comparative analysis of NLR sequences between resistant and susceptible groups. (**B**) AgRenSeq performs NLR enrichment in a natural diversity panel and conducts k-mer-based association study to identify NLRs linked to resistance. Created in BioRender. Liu, Z. (2025) https://BioRender.com/i2jkvvv (accessed on 20 January 2026).

Mutagenesis with *R* gene enrichment sequencing (MutRenSeq) employs EMS mutagenesis to generate loss-of-resistance mutants from a resistant line, enabling comparative RenSeq analysis between resistant and susceptible groups (Figure 2A). This facilitates direct cloning of causal NLRs from complex genomes without further fine mapping [32]. This has resulted in the identification of multiple key wheat genes against major diseases, including *Yr7*, *Yr5*/*YrSP*, *Sr22*, *Sr45*, and *Sr65* [32,55,56]. Compared with large-scale GWAS phenotyping, MutRenSeq requires smaller populations, as screening for loss-of-resistance mutants is simpler. However, MutRenSeq is best suited for revealing qualitative resistance genes, as the identification of minor quantitative resistances is difficult due to the absence of genetic diversity from the single parental line.

Aassociation genetics with *R* gene enrichment sequencing (AgRenSeq) extends RenSeq beyond single-gene analyses to the discovery of quantitative resistance loci. By coupling RenSeq with short DNA fragment k-mer-based association genetics across natural populations, AgRenSeq identifies genotype-phenotype associations through statistical analysis of k-mer frequency differences among different accessions [33] (Figure 2B). This improves the detection of quantitative and minor resistance genes in crops with diverse germplasm. As a proof-of-concept, AgRenSeq identified *Sr33* and *Sr46* against *Puccinia graminis* f. sp. *tritici* from a panel of 195 wild wheat relatives [13,33,57]. The combination of AgRenSeq with SMRT across 117 *Solanum tuberosum* L. (potato) accessions further identified multiple resistance-causing genes against late blight and *Globodera pallida* [54].

Although MutRenSeq and AgRenSeq generate focused candidate lists, they are limited to NLRs, overlooking mutations in non-NLR genes. Alternatives such as mutant chromosome flow sorting and sequencing (MutChromSeq), which uses flow-sorting and sequencing of chromosomes containing pre-mapped resistance loci [58,59], and mutagenesis and transcriptome sequencing (MutRNA-Seq), which detects causal mutations through transcript sequencing [60], offer complementary approaches. However, MutChromSeq is technically demanding, and RNA-seq-based methods show lower sensitivity and accuracy than RenSeq [61]. Overall, MutRenSeq and AgRenSeq demonstrate strong potential for identifying qualitative and quantitative resistance genes, and, together with other RenSeq-derived strategies, serve as effective alternatives to traditional genetic approaches for NLR discovery.

The success of RenSeq-derived approaches relies heavily on advances in NLR annotation. In the early stages of RenSeq, the Pfam database was used to annotate contigs generated from sequencing. With the development of specialized tools such as NLR-Parser and NLR-Annotator, NLR annotation has become deeply integrated into the RenSeq workflow. Both MutRenSeq and AgRenSeq employ NLR-Parser at the initial stage to screen NLR genes from the genomes of related species for probe design, and again after sequencing and assembly to annotate candidate NLR sequences. MutRenSeq further performs double annotation checks on differentially expressed NLR candidates, while AgRenSeq also filters k-mers for NLR signatures [32,33]. The HIgh-throughput SMRT-AgRenSeq-d Snakemake (HISS) workflow proposes a detailed pipeline that uses NLR-Annotator to efficiently identify novel resistance genes from raw RenSeq sequencing data [40]. Here, we also highlight the critical role of NLR annotation tools throughout the RenSeq process, from probe design in the early stage, to contig annotation in the middle stage, and to subsequent validation of NLR candidates. This is particularly important in studies that leverage sequencing data from related species to investigate NLR genes in non-model plants.

### 2.2. Omics-Based Unbiased Identification of Immunity-Related Genes

Instead of following the conventional phenotype-to-genotype route, novel experimental techniques can identify candidate immunity genes based on their functional relationship with disease resistance. By exploiting functional association to resistance phenotypes in place of genetic association, an untapped pool of candidate immunity genes without extensive natural genetic variation can be revealed [62,63]. Additionally, generating candidate gene lists separate from natural variation becomes more important due to the increasing amenability of gene editing for validating and applying such immunity genes in resistance breeding [64,65]. The independence of natural genetic variation also improves the accessibility of such methods for academic labs by eliminating the need for large-scale population development and extensive phenotyping.

A straightforward and powerful way to identify candidate genes based on experimental correlation to disease resistance is by quantitatively comparing gene products between susceptible and resistant genotypes. Traditionally, such omics approaches, including transcriptomics, proteomics, and metabolomics, have been extremely valuable for identifying major pathways and processes involved in the pathogen infection response [66,67,68]. Comparing the RNA, protein and metabolite levels between a resistant and susceptible plant yields an unbiased list of molecular differences that can cause resistance. The untargeted omics approaches can therefore identify all types of immunity gene types. Additionally, by relying on functional relationships instead of genetic association to resistance phenotypes, omics approaches reveal both major and minor quantitative resistance loci, which further enhances resistance breeding [14]. Nevertheless, the unbiased nature of omics approaches, along with the independence of genetic association, forms a double-edged sword. As resistance mechanisms involve many changes on the RNA-, protein-, and metabolite-level, functional validation of the vast number of candidate genes obtained by traditional RNA-sequencing (RNA-seq), proteomics, and metabolomics is typically unfeasible [69,70,71]. Moreover, in contrast to genetic methods, the functional correlation between differentially expressed candidate genes and the disease phenotype is often not causal, which increases the dependence on functional validation [72]. Technological omics advances are therefore required to go from revealing general resistance pathways to generating a shorter, more valuable list of candidate immunity genes feasible for validation. The rapid emergence of single-cell and spatial omics, as well as plant metabolomics, can improve the focus towards immunity-related genes [67,73]. In the following sections, the most promising omics tools for identifying more relevant and feasible lists of candidate immunity genes are discussed.

#### 2.2.1. Finetuned Transcriptomics: scRNA-seq

Transcriptome-wide RNA-seq is the most established omics approach for studying pathogen infection and resistance mechanisms [73]. Due to advancements in major sequencing technologies and genome annotation, RNA-seq is a high-throughput and widely applicable method that enables sensitive RNA quantification. The extensive catalogue of commercial RNA-seq providers and its reduced cost also contribute to the method’s accessibility [73,74]. As RNA-seq usually results in hundreds to thousands of differentially expressed genes, of which only some contribute to the phenotype, RNA-seq is typically applied to reveal major resistance-related pathways instead of aiming to discover individual candidate genes for validation [66,72]. Moreover, pathogen infection in plants is a heterogeneous process, as infection responses differ greatly between tissue types and different infection phases can exist in parallel [75,76]. General transcriptomic changes thus represent the average disease response across different tissues and infection stages [75].

In contrast to untargeted comparative RNA-seq, single-cell RNA-sequencing (scRNA-seq) enables the analysis of cell-specific changes during pathogen infection, which greatly improves the signal-to-noise ratio [34]. Single-cell transcriptomics first requires generating protoplasts from infected tissue, after which the protoplasts are merged with uniquely barcoded beads carrying poly-dT probes via microfluidics. After cell lysis, on-bead reverse transcription is then performed on thousands of individual cells to generate a single-cell cDNA library [73]. As differentially expressed genes identified by scRNA-seq have an increased specificity for immunity-related processes, scRNA-seq can generate shorter and less diluted candidate immunity gene lists that are feasible for functional validation [34,73]. Recently, multiple studies have demonstrated this by identifying and functionally validating novel immunity genes across several major crops using scRNA-seq. Notable examples include the discovery of antiviral roles for *OsJiPR10* against rice blast disease, *ZmChit7* against southern corn rust, and *OsHKT9* against rice black streaked dwarf virus, which was confirmed in overexpression (*ZmChit7*) or knockout (*OsHKT9* and *OsJiPR10*) lines [77,78,79]. Although mutant lines are preferred for absolute validation, the combination of scRNA-seq and transient silencing or overexpression assays can simultaneously reveal multiple antiviral and proviral genes. In this way, four *Zea mays* L. (maize) genes (*ZmSAM-Mt*, *ZmCDF1*, *ZmCC7*, and *ZmPLT1*) [80] and two *Glycine max* (soybean) genes (*GmGSTU23* and *GmGSTU24*) [81] were shown to contribute to resistance against mosaic viruses.

The independence of extensive population and genomic annotation requirements facilitates the use of scRNA-seq to uncover resistance-related genes for a wide variety of crops [82,83,84]. Liang. et al. performed scRNA-seq of *Hevea brasiliensis* (rubber trees) followed by overexpression of candidate genes in *Nicotiana benthamiana* (tobacco) to reveal the potential of the NLR gene *HbCNL2* to confer powdery mildew resistance [82], while Zang et al. similarly showed that *ScNPR3* might increase the susceptibility of *Saccharum* sp. (sugarcane) to smut disease [83]. By combining scRNA-seq and candidate gene silencing in *Rosa* sp. (roses), Li et al. identified two genes involved in susceptibility and one in resistance to *Botrytis cinerea* [84]. Broader use of scRNA-seq to study plant-pathogen interactions still encounters multiple methodological challenges, such as the need for fluorescent reporter lines, complex isolation of infected protoplasts in recalcitrant crops, and challenging detection of low-abundant immunity transcripts [34]. Nevertheless, these studies position scRNA-seq as a widely applicable and powerful approach for generating a focused list of candidate immunity genes suitable for validation.

Due to the loss of spatial resolution and the challenging sample preparation of scRNA-seq, complementing single-cell transcriptomics with spatial transcriptomics of tissue sections can increase the power of scRNA-seq to reveal resistance mechanisms [73,85]. While most examples of spatial transcriptomics itself or combined single-cell and spatial transcriptomics cover fundamental questions of plant immunity [73,85], its ability to pinpoint specific genes contributing to disease resistance has recently been demonstrated [78]. We also envision integration of single-cell and long-read RNA-sequencing to simultaneously provide more reliable genome annotations, which is particularly important for non-model crops and the accurate annotation of complex NLR genes [86]. Additional benefits are the ability to study the role of alternative splicing isoforms and long non-coding RNA transcripts in disease resistance [87]. However, long-read RNA-seq has greater input requirements than conventional short-read RNA-seq, underscoring the need for improved sample preparation techniques for single-cell transcriptomics in plants.

#### 2.2.2. Increased Relevance of Proteins: Proteomics

Since only proteins represent the end products of regulatory networks, and due to the divergence between RNA and protein level changes [88], proteomics is expected to be more accurate than RNA-seq for explaining resistance mechanisms [71]. Especially in well-studied crops, the strong functional relevance of differentially abundant proteins can enable direct identification of immunity genes rather than solely mapping the main resistance pathways [89]. This enhanced relevance of proteomics, however, comes with reduced feasibility and accessibility due to resource-intensive mass spectrometry usage, complicated data analysis, and the requirement of thoroughly annotated proteomes [90,91]. Additionally, despite essential advances, including label-free quantitative proteomics, the coverage of proteomics experiments is more restricted due to sample preparation and mass spectrometry sensitivity limitations [68,90]. Based on these considerations, using proteomics instead of transcriptomics to screen for genetic resistance might be more valuable, provided that the plant-pathogen system is relatively well-studied and sufficient expertise on mass spectrometry is available [69].

While comparative proteomics has been essential for revealing general resistance mechanisms, the increased relevance of proteomics does not necessarily result in a greatly reduced number of candidate genes compared to RNA-seq [68]. This still makes the selection of candidate genes for validation severely challenging. Notably, the high relevance of comparative proteomics combined with well-described protein functions for major crops can facilitate the targeted identification and, consequently, feasible validation of the underlying resistance genes. In this way, Gupta et al. identified *OsArg1* as a putative regulator of resistance against *Xanthomonas oryzae* pv. *oryzae*, and generated overexpression mutants in *Oryza sativa* L. (rice) to confirm this [89]. In combination with more transient validation approaches, comparative proteomics identified multiple genes contributing to resistance against *Botrytis cinerea* in roses [92], *Fusarium graminearum* in maize [93], and *Magnaporthe oryzae* in rice [94]. Yet, comparative proteomics directly followed by functional validation might only be attainable for a limited number of plant-pathogen combinations, and mainly those where considerable functional genomics data or other prior knowledge is available to filter candidate genes.

Single-cell proteomics holds great promise for generating more focused lists of candidate immunity genes; however, its application is currently hindered by relatively high detection thresholds and limited throughput [73]. Alternatively, tissue- and organelle-specific proteomics has facilitated the more targeted identification of actual immunity genes while circumventing sensitivity limitations [71]. For example, analysis of the leaf epidermis proteome in barley followed by candidate silencing revealed that the TLP5 protein increases susceptibility against *Blumeria graminis*-induced powdery mildew [95]. Likewise, comparative proteomics of *Pyrus* sp. (pear) apoplasts suggested that PbrGlu1 contributes to resistance against *Colletotrichum fructicola* infection [96]. While (organelle-specific) proteomics is clearly able to pinpoint immunity genes, it remains imperative to consider the balance between the greater relevance of proteomics and the broader applicability of transcriptomics. Another essential question is whether and when single-cell proteomics could have a similar rise as single-cell RNA-seq for identifying genetic resistance.

#### 2.2.3. From Biomarkers to Causal Genes: Metabolomics

Secondary metabolites are, along with proteins, the essential mediators of biotic stress responses due to their involvement in pathogen surveillance, signal transduction, and anti-microbial activities [67,97]. Because metabolomics can accurately reflect the physiological state of the plant, its close link to the phenotype makes metabolomics exceptionally relevant for studying plant-pathogen interactions [67]. Hence, by providing critical insights into how plants reprogram their metabolism during infection and how pathogens manipulate host metabolic pathways, metabolites driving resistance and susceptibility can be discovered. Such metabolites can then be directly used as resistance biomarkers [69]. However, the lack of comprehensive plant secondary metabolite databases combined with substantial chemical diversity and technical variation complicates the application of quantitative metabolomics [67,69]. Nevertheless, many qualitative comparative metabolomics approaches have identified multiple secondary metabolites that regulate disease resistance. For example, analysis of metabolic alterations during *Phytophthora infestans* infection in six potato cultivars revealed four steroidal saponins that have strong antioomycete activity [98], whereas a similar approach found that increased levels of another saponin, bayogenin 3-O-cellobioside, improve resistance against rice blast disease [99]. By performing metabolomics of the leaf surface of a potato wild relative species, the fatty acid lysophosphatidylcholine 17:1 was shown to contribute to resistance against *Phytophthora infestans* [100]. While metabolite validation was mainly based on in vitro viability assays rather than on mutant lines, metabolomics can reveal compounds with great potential as biomarkers for resistance breeding. Moreover, as metabolomics does not depend on well-annotated genomes and transcriptomes, it is also suitable for identifying metabolites regulating resistance in less well-studied crops. Promising examples include its application for *Actinidia chinensis* L. (kiwi) [101] and *Beta vulgaris* (sugar beet) [102] disease research, but candidate filtering and validation remain challenging for non-model crops.

The use of metabolomics to identify immunity genes that actually underlie metabolite-based resistance could be more valuable than just employing resistance-related metabolites as resistance biomarkers for breeding [103]. However, secondary metabolites are often not unambiguously connected to single genes, which severely complicates the identification of underlying resistance genes [97]. Due to this bottleneck, few studies have combined the metabolomic identification of resistance-related metabolites with functional validation of key genes in the metabolite’s pathway [103,104]. Due to its simple yet accurate identification of differentially expressed genes, the integration of RNA-seq and metabolomics enables connecting resistance-related metabolites to candidate immunity genes in the metabolite’s pathway. In this way, the flavanone synthesis-related gene *OsF3’H* was identified, for which knockout lines displayed reduced susceptibility against both brown planthopper and blast disease in rice [105]. Metabolo-transcriptomics followed by transient validation assays further revealed that the auxin receptor *TaTIR1* reduces resistance against *Fusarium graminearum* in wheat [106] and that the flavonoid biosynthesis transcription factor OeWRKY40 contributes to *Alternaria alternata* resistance in *Olea europaea* (olive) trees [107], thereby demonstrating the potential of integrated metabolo-transcriptomics to identify immunity genes.

In recent years, multi-omics data has increasingly been integrated with GWAS results to narrow down the list of candidate genes. QTL intervals identified by GWAS often span a large number of genes, making subsequent fine-mapping laborious and time-consuming. Comparative omics analyses can be used to identify differentially expressed genes or pathways under infection conditions, which then serve as effective filters for prioritizing genes within GWAS candidate regions [23,24,25]. For example, Wang et al. first used GWAS to identify a major QTL for *Verticillium wilt* resistance in *Gossypium* sp. (cotton) spanning a 902.6 kb region containing 95 genes. Through differential expression analysis based on transcriptomic data, they further narrowed the candidates and identified *GhAMT2* as the core gene, which was subsequently functionally validated [25]. This type of prioritization strategy currently represents a common and powerful form of “GWAS-multi-omics” pipeline in plant research. In essence, it bridges phenomics, genomics, and transcriptomic/proteomic/metabolomic data to refine plant resistance gene selection.

### 2.3. Interactomics to Unravel the Molecular Plant-Pathogen Interactions Causing Resistance

Molecular interactions between plants and pathogens play a pivotal role in infection and plant immunity, occurring across multiple levels, including protein–protein interaction (PPIs), RNA-protein interactions, and DNA-protein interactions. For instance, distinct peptides of bacterial flagellin are recognized by different pattern recognition receptors [108]. Additionally, several experimentally validated NLRs have been shown to directly interact with pathogen effectors [109]. The eukaryotic translation initiation factor 4E (eIF4E) interacts with viral genomic RNA to facilitate viral RNA translation for multiple plant viruses [110]. Furthermore, pathogen-derived transcription activator-like effectors induce the expression of sugars will eventually be exported transporters (SWEET) genes, promoting sugar efflux from the phloem into the apoplast, thereby enhancing host susceptibility by supplying nutrients to pathogens [111]. Although not all molecular interactions regulate plant disease resistance, many serve as critical immunity nodes and represent promising candidate immune genes. Interactomics approaches driven by biochemical methods offer advantages over traditional genetic approaches, such as lower resource demands and shorter experimental timelines. Importantly, interactomics also provides valuable insights into the molecular mechanisms of disease resistance. Compared to comparative proteomic profiling, interactomics can yield a more targeted list of candidate genes and improved detection sensitivity for low-abundance proteins [71]. Here, we primarily review the emerging experimental techniques for studying protein–protein interaction and RNA-protein interactions in plant immunity, as well as novel computational methods for predicting protein–protein interaction.

#### 2.3.1. Protein–Protein Interactions: Proximity Labelling

Traditional methods for screening protein–protein interaction, such as yeast two-hybrid, affinity purification mass spectrometry, and co-immunoprecipitation mass spectrometry, have been applied in the field of plant-pathogen interactions for years [112,113,114,115]. These well-established and widely adopted techniques, however, exhibit inherent limitations. For instance, yeast-based systems are heterologous and often associated with high rates of false positives, while both affinity purification and co-immunoprecipitation mass spectrometry require specific antibodies. Moreover, none of these methods are well-suited for detecting weak or transient interactions. Recent advances in proximity labeling technologies offer promising opportunities to overcome these shortcomings.

**Figure 3:**
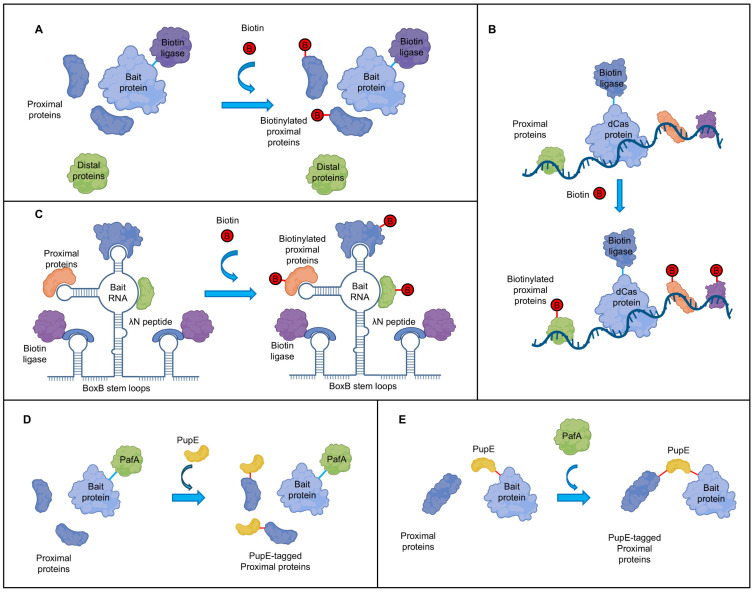
Proximity labeling methods in protein–protein interaction and RNA-protein interactions. (**A**) Proximal proteins are biotinylated by a biotin ligase fused to the target protein. (**B**) Proximal proteins are biotinylated by a biotin ligase, which is fused with a dead Cas9 protein and directed to the bait RNA. (**C**) The bacteriophage λN peptide and BoxB stem-loop structure are fused to a biotin ligase and RNA of interest, respectively, and proteins proximal to the RNA are biotinylated. (**D**) PafA is fused to a bait protein and mediates PupE modification of prey protein. (**E**) PupE is fused to a bait protein, and PafA mediates the covalent attachment of PupE to prey proteins. Created in BioRender. Liu, Z. (2025) https://BioRender.com/okj1dka (accessed on 20 January 2026).

The principle of proximity labeling involves fusing an enzyme with proximity labeling functionality to the bait protein, usually a biotin ligase that biotinylates the proteins close to the bait. The biotinylated protein interactors are subsequently enriched using streptavidin magnetic beads and identified through mass spectrometry, enabling comprehensive analysis of protein interactions and the local protein environment surrounding the target (Figure 3A). Since the initial development of the BioID biotin ligase, the proximity labeling toolbox has greatly expanded with various enzymes optimized for labeling efficiency and cell compatibility, including peroxidases (e.g., APEX, APEX2, and HRP) and biotin ligases (e.g., BioID2, BASU, TurboID, miniTurbo, Split-BioID, and Split-TurboID) [116]. Biotin ligases are generally preferred over APEX for in vivo studies by eliminating the need for toxic H_2_O_2_ and biotin-phenol substrates [71].

The versatility of proximity labeling has been demonstrated in numerous studies of plant-pathogen interactions, particularly when pathogen effectors are used as bait proteins, leading to the identification of several effector interactors. For instance, BioID was employed by Conlan et al. to identify the interaction partners of AvrPto, a *Pseudomonas syringae* effector protein, discovering two *S* factors APK1 and TOM1 and demonstrating an important role for the central immune component RIN4 [117]. Zhang et al. exploited the superior performance of TurboID over BioID to explore the interactome of a tobacco mosaic virus resistance protein in *Nicotiana benthamiana*, leading to the discovery of susceptibility factor UBR7 [118]. Later, TurboID was also used to elucidate the putative mechanism behind maize susceptibility to the *Ustilago maydis* effector UmSee1 [119]. In addition to susceptibility factors, interactions between the NLR protein family and pathogen effectors have also been detected by proximity labeling, such as for barley MLA13 [120]. These examples demonstrate the feasibility of using proximity labeling with pathogen effectors as bait proteins to screen for their immunity-related host targets.

Beyond these achievements, proximity labeling has demonstrated potential in several more specialized areas. Firstly, the incorporation of additional localization signals has enabled spatially resolved proximity labeling. By engineering labeling enzymes with organelle-specific signals, these enzymes can be directed to specific subcellular compartments, thereby achieving organelle-level resolution and significantly reducing background noise [121]. Another effective strategy for minimizing background interference is involving proximity labeling in protoplast systems. For instance, Lin et al. successfully employed the BioID system in rice protoplasts to identify proteins interacting with OsFD2, a key regulator of vegetative growth [122]. Finally, the utility of proximity labeling in studying plant-pathogen interactions is now expanding to the level of RNA-protein interactions. This can be accomplished, for example, by fusing a biotin ligase to an RNA-binding protein (e.g., λN peptide) that recognizes a specific RNA aptamer (e.g., BoxB stem-loop) [123] or by leveraging a catalytically dead Cas (dCas) protein to guide the ligase to a target RNA sequence [124,125] (Figure 3B,C). While these RNA-protein proximity labeling approaches have been successfully established in human viral research, such as Zika virus [125], they hold considerable promise for future applications in plant virology.

Although recent advancements in proximity labeling have greatly expanded our ability to investigate protein–protein interaction, several critical limitations remain to be addressed. Major concerns are the potential malfunction of the bait protein caused by fusion with large proximity labeling enzymes (typically 20–54 kDa), as well as cytotoxic effects resulting from excessive levels of biotin or hydrogen peroxide used as substrates [126]. The duration of biotinylation also requires careful optimization, since proximity labeling labels all proteins within the enzyme’s catalytic radius more than genuine interactors. Extended labeling periods may therefore increase nonspecific modifications and lead to false positives. While some studies suggest that proximity labeling performs well for detecting membrane-associated protein interactions, others hold the opinion that membrane structures can hinder biotinylation by limiting the diffusion of reactive intermediates [117,127]. In addition, the presence of endogenously biotinylated proteins, which are particularly abundant in plant chloroplasts, can interfere with experimental outcomes [127].

As an alternative, pupylation has emerged as a promising approach. This system utilizes prokaryotic ubiquitin-like protein ligase A (PafA) to covalently attach prokaryotic ubiquitin-like protein (Pup) to target proteins, thereby labeling proximal interaction partners [128,129,130] (Figure 3D,E). Unlike biotinylation, pupylation is absent in plants and does not require exogenous substrate addition, effectively reducing background interference caused by endogenous biotin. As a proximity labeling-like strategy, pupylation-based methods have recently been introduced into plant research [131,132,133], thereby holding potential for examining plant-pathogen interactions.

#### 2.3.2. Computational Prediction of Protein–Protein Interactions

The computational prediction of protein–protein interaction is becoming a powerful and rapid way to improve the selection of candidate interactors, which can greatly increase the focus and decrease the scale of interactomics experiments. Conventional PPI prediction relies on sequence homology, such as interolog (conserved interacting orthologs) and domain-based approaches, which infer putative interactions between homologous proteins from known PPI datasets [134,135]. However, experimentally validated plant-pathogen PPI data remain limited: the PHI-base contains only 414 plant-pathogen pairs, while the plant-specific PathoPlant database includes 351 [136]. This scarcity reduces the sensitivity of ortholog- and domain-based prediction. Incorporating cross-species templates can improve sensitivity, but often increases false positives due to heterogeneous data [137]. To address this, machine learning has been applied to evaluate prediction reliability [138,139]. For instance, candidate PPIs between Magnaporthe grisea and rice were identified using interolog- and domain-based approaches, followed by support vector machine-based validation [138].

Yet, due to cross-species integration and extensive domain diversity, these methods frequently generate extensive PPI lists, often containing thousands of targets that are difficult to interpret and verify [134,135]. More recently, machine learning models have been used to predict PPIs from multidimensional features. For example, Deep-HPI-pred employs a multilayer perceptron trained on seven network centrality measures to predict PPIs. Applied to *Citrus* L. (citrus)-*Candidatus Liberibacter asiaticus*, the causal agent of citrus greening disease, and Arabidopsis-*Pseudomonas syringae* pairs, it achieved over 90% accuracy in classifying interacting and non-interacting proteins [140]. Beyond sequence-based features, topological properties of PPI networks are increasingly incorporated [10,137,141]. Furthermore, ensemble models combining Random Forest, Support Vector Machine, and Artificial Neural Network via soft voting outperform individual models in predicting plant-pathogen interactions [136]. These models can produce fewer and more reliable predicted interactions.

Except relying on sequence features, protein structure prediction tools such as AlphaFold-Multimer [36] and RFdiffusion [142] further facilitate PPI modeling by simulating spatial conformations of protein complexes and assessing structural reliability [36,109]. For example, AlphaFold-Multimer predicts plant-pathogen PPIs by modeling interactions between pathogen effectors and plant resistance proteins, evaluating reliability using 80% interface predicted template modeling score and 20% template modeling score [36]. Alternatively, Haley et al. employed RFdiffusion to generate hypothetical binders for *Magnaporthe oryzae* effectors and screened the rice proteome for structurally similar candidates [142]. These structure-based tools thus hold great potential to advance research into plant-pathogen interactions and to accelerate the experimental identification of immunity genes.

Despite these improvements, most models above are based on supervised learning, relying on pre-existing data labels. In this way, the limited number of experimentally confirmed plant-pathogen PPIs complicates the computational discovery of novel PPIs. Although unsupervised and semi-supervised methods remain computationally demanding, they hold potential to expand understanding of plant PPIs, particularly when PPI templates are scarce and omics datasets are high-dimensional and redundant [143,144]. Moreover, predicting protein dynamics and post-translational modifications from such models remains debated [145,146].

Currently, most computational predictions remain at the stage of generating candidate lists, often generating thousands of candidate genes that are impractical to validate experimentally [134,135]. However, these predictions can be effectively integrated with upstream experimental protein interaction assays. Proteins identified through proximity labeling or AP-MS screens, for example, can be filtered using computational prediction to obtain a more focused and higher-confidence candidate list. Conversely, candidate lists derived from computational predictions can also be coupled with the high-throughput CRISPR screening approaches discussed below, enabling efficient functional validation at scale.

#### 2.3.3. RNA-Protein Interactions: Viral Ribonucleoprotein Isolation

RNA viruses constitute a large group of plant pathogens. While many viral proteins have been characterized through protein–protein interaction studies, viral RNA-binding proteins (RBPs), which support critical processes including virus replication, stability, transmission, and evasion of host immune responses [147,148] remain poorly understood in plants. Given the rapid technological advances in the investigation of RNA-protein complexes (RNPs) [149,150] and their expanding applications in human virology [151,152,153,154], viral RNA-binding proteins have emerged as promising candidates for immune-related gene discovery.

Various innovative approaches are available for investigating viral RNA-protein interactions. Native RNA pulldown is a well-established technique for studying RNA-protein interactions. It has been widely applied in human pathogen research, including studies on hepatitis, Zika, and dengue virus [155,156]. However, its application in plant-virus systems remains limited. A related example is the work by Incarbone et al., who used the GFP-tagged dsRNA-binding protein B2:GFP to pull down dsRNA and associated proteins during tobacco rattle virus infection in planta. Mass spectrometry analysis identified both viral proteins and host proteins, leading to the discovery of Arabidopsis DRB2 as a broad-spectrum antiviral factor [157]. Compared to native RNA pull-down, alternative methods for studying RNA-protein complexes using UV crosslinking or formaldehyde have been continuously refined, offering advantages in physiological conditions for detecting weak and transient interactions.

Typical in vivo research on plant virus RNPs encompasses cross-linking RNA with its adjacent proteins, isolation of specific RNPs, and mass spectrometry proteomic profiling. RNP isolation is commonly performed with the acid guanidinium thiocyanate-phenol-chloroform method [158], in which RNPs accumulate at the interphase between the upper aqueous phase and the lower organic phase. In this way, multiple recent techniques such as orthogonal organic phase separation (OOPS), plant phase extraction (PPE), and silica-based acidic phase separation (SAPS) have elucidated the plant RNA-binding proteome (RBPome), as well as its changes during pathogen infection [159,160,161,162]. However, similar to proteome profiling, the entire RBPome can yield an extensive list of candidates and can be less accessible. In contrast, a more focused viral RNA interactome isolation could result in the more targeted identification of RBPs interacting with viral RNA. Approaches to obtain this typically employ biotinylated antisense oligonucleotide probes that hybridize with target viral RNA, followed by streptavidin bead pulldown to isolate the RNA along with its cross-linked proteins. Examples from human virus studies include the chromatin isolation by RNA purification-mass spectrometry (ChIRP-MS), RNA antisense purification and quantitative mass spectrometry (RAP-MS), and hybridization purification of RNA-protein complexes followed by mass spectrometry (HyPR-MS) methods, which have been successfully applied in studies of SARS-CoV-2 and HIV-1, identifying hundreds of viral RNA-binding proteins and both positive and negative regulators for virus infection [152,154,163]. More recently, targeted RNase H-mediated extraction of crosslinked RBPs (TREX) and selective RNase H-mediated interactome framing for target RNA regions (SHIFTR) target RNA hybridizes with antisense DNA probes to form RNA-DNA duplexes, which are then specifically degraded by RNase H. This process selectively releases RBPs associated with the targeted RNA into the organic phase, thereby greatly enhancing the specificity and enrichment of RBPs bound to the desired RNA segment [164,165]. In plants, a related example of viral RNA-specific RBP identification is the probe-based pulldown of citrus yellow vein associated virus RNPs from infected *Cucumis sativus* (cucumber) phloem sap without cross-linking [166]. Additionally, cross-linking and antisense probe pulldown recently revealed the interactome of AtGRP7, an abundant circadian clock-regulated messenger RNA, in Arabidopsis [167]. However, identifying specific RNA interactomes in plants remains challenging due to the complexity of plant tissues [161,168]. Although these methods are not yet frequently applied in plants, the abundance of human virus research suggests that revealing the interactome of plant virus RNA holds great potential to identify both proviral and antiviral immunity genes.

## 3. High-Throughput Approaches to Validate Candidate Immunity Genes

As targeted resistance breeding using causal variants is preferred over breeding quantitative trait loci, the acceleration of crop breeding mainly depends on generating more focused candidate gene lists and improving the subsequent functional validation approaches [29]. Although the above methods already produce more relevant candidate immunity gene lists, the scale of subsequent validation remains a bottleneck for identifying the causal gene [37]. For example, despite extensive pipelines combining GWAS and multi-omics integration, Knoch and colleagues identified multiple candidate genes for early vegetative growth of canola that still await functional validation [169]. Similarly, many studies applying cutting-edge methods to identify candidate immunity genes have yet to progress into validation [17,37]. The use of relevant candidate gene lists to identify specific functional variants thus relies heavily on improved and scalable validation strategies. Recently, several CRISPR/Cas-based approaches with high-throughput validation potential have been applied to plants, including pooled CRISPR screening, CRISPR silencing and CRISPR activation [65,170]. Moreover, the use of CRISPR for validation is encouraged by the trend toward deregulating non-transgenic CRISPR-edited crops, which could facilitate the use of CRISPR-generated variation in resistance breeding [64].

### 3.1. Advanced CRISPR Approaches to Validate in Planta

#### 3.1.1. Large-Scale Generation of Loss-of-Function Mutants: CRISPR Multiplexing and Pooled CRISPR Screens

CRISPR/Cas-based genome editing to generate knockout lines is the most conventional and powerful approach to confirm the causal relationship between candidate genes of any class and disease resistance. In contrast to its established use for characterising individual genes, high-throughput genome editing in plants for validating candidate genes is hindered by its low transformation efficiencies, cumbersome regeneration, and extensive facilities required for examining large mutant populations [37,65,171]. To increase the combinatorial potential of CRISPR/Cas9 editing, multiplex CRISPR and pooled CRISPR screening are powerful innovations [171,172].

Multiplex CRISPR, where multiple guide RNAs are arrayed on a single vector to generate different combinations of edited target genes, is mainly used to examine functional redundancy or genetic interactions between related genes [172]. The strength of multiplex CRISPR for validating immunity genes, therefore, lies in the concurrent targeting of multiple orthologs to identify resistance phenotypes otherwise masked by genetic redundancy within gene families, a phenomenon frequently observed in crops [171]. Additionally, bioinformatic tools such as MINORg can design non-specific guide RNAs to target several related sequences at once; this significantly reduces the number of guide RNAs required to cover a gene family [173]. A landmark example was the multiplex editing of promoter regions for all three OsSWEET sucrose transporter genes in rice, which resulted in broad-spectrum resistance to bacterial blight [174]. Hong and colleagues also applied a multiplex CRISPR/Cas9 system in tomato to reveal that knockout of both the miR482b and miR482c microRNAs is required for strong resistance against *Phytophthora infestans* [175].

**Figure 4:**
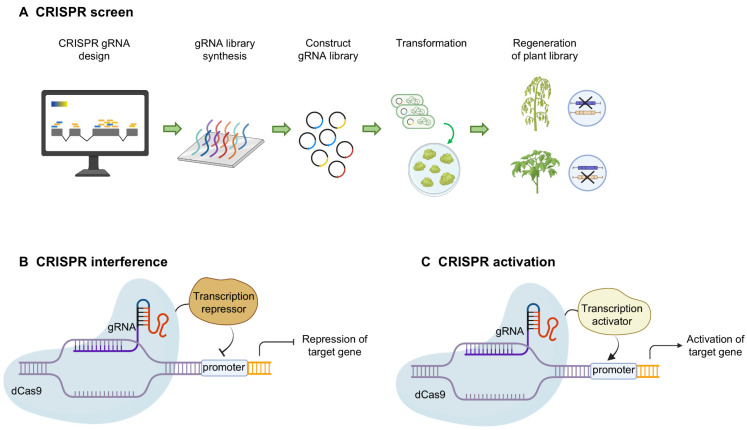
Schematic diagram of candidate immunity gene validation by CRISPR gene editing. (**A**) In CRISPR screens, a gRNA library is designed and synthesized, followed by pooled transformation of plant cells. After regeneration, a large plant population containing many single gene knockout lines is obtained that can be screened for pathogen resistance. (**B**) The principle of CRISPR interference to stably inhibit target gene expression using a catalytically dead Cas9 fused to a transcription repressor. (**C**) The principle of CRISPR activation to stably induce target gene expression using a catalytically dead Cas9 fused to a transcription activator. Created in BioRender. Liu, Z. (2025) https://BioRender.com/qoawsxq (accessed on 20 January 2026).

By pooling many individual Cas9-guide RNA constructs for high-throughput plant transformation, generating a large population of single knockout mutant lines has become conceivable (Figure 4A). Mainly depending on the available workforce and growth facilities, pooled CRISPR/Cas9 screens with comprehensive guide RNA libraries have generated collections of more than a thousand mutant crop lines [171]. Based on this, pooled CRISPR screening is also suitable for the functional validation of substantial lists of unrelated candidate immunity genes, as obtained through omics approaches [176]. By generating a pool of 969 guide RNAs that target 502 differentially expressed genes during aphid infestation in cotton, the broad-spectrum insect resistance gene *GhMLP423*, as well as the mechanism behind it, was revealed [177]. Another pooled CRISPR screen in cotton of the calcium-dependent protein kinases demonstrated that the homologs GhCPK33 and GhCPK74 confer resistance against multiple chewing pests [178]. Through an innovative approach termed PCR fragment-length markers for distinguishing guide RNAs (FLASH), Chen et al. accelerated the process of guide RNA sequencing to significantly enhance the efficiency of CRISPR screening in rice. In this way, a mutant library consisting of 710 out of 1072 receptor-like kinases was generated, and for nine of them, decreased resistance against *Magnaporthe oryzae* was observed [179]. To further expand the use of pooled CRISPR screens for validating candidate gene lists, improved integration of all parameters that determine a CRISPR screen’s efficiency is essential. Hence, to obtain an optimal CRISPR screen design, more prior knowledge is required on the selected editing tool and transformation format, the number of target genes, the mutagenic efficiency and the guide RNA construction accuracy [171]. A recent detailed guideline on the design of pooled CRISPR screens in plants is described by Van Huffel et al. [172]. In addition, as high-throughput transformation only indirectly reduces the workload of other steps, such as guide RNA design, tissue regeneration and disease phenotyping, through the parallelisation of work, CRISPR screening remains a resource-intensive and often rate-limiting validation approach.

#### 3.1.2. CRISPR-Based Transcriptional Modulation of Candidate Genes: CRISPRi and CRISPRa

Instead of generating a loss-of-function mutant, transcriptional repression can also be employed for high-throughput validation of candidate immunity genes. This is especially useful to limit yield-related trade-offs or when (combinations of) genomic knockouts are lethal [65]. Conventional silencing approaches in plants, including RNA interference or virus-induced gene silencing, are more scalable than stable transformation. However, their low silencing efficiency is often insufficient to reveal phenotypes, and the small interfering RNA pathway frequently generates off-target effects. The application of a novel silencing method based on CRISPR-dead Cas9 constructs fused to transcriptional silencing domains is showing promise in plants [180]. The CRISPR interference (CRISPRi) approach exploits the exceptional target specificity of computationally designed guide RNAs to improve silencing selectivity, while the coupling of dead Cas9 to plant transcriptional repression domains such as SRDX and ZAT10 improves the silencing efficiency [180] (Figure 4B). Until now, CRISPRi has mainly been applied for functional genomics studies in plants and not for high-throughput screening or the modulation of immunity gene expression levels [170,180]. Nevertheless, several CRISPRi screens with extensive guide RNA libraries in other hosts suggest that CRISPRi could become an alternative to regular CRISPR screening for high-throughput immunity gene validation in plants with less pleiotropic effects [170,181].

By coupling a catalytically dead Cas9 to a transcriptional activation domain in CRISPR activation (CRISPRa) assays, high-throughput overexpression of target genes can be achieved (Figure 4C). Similarly to CRISPRi, CRISPRa can circumvent the lethality and yield reductions often observed for genomic knockouts. Additionally, it also has selectivity and reproducibility advantages over conventional Agrobacterium overexpression approaches because of the great target specificity of guide RNAs and the strong transcriptional activation by artificial VPR or SunTag domains [170,171]. Moreover, gain-of-function approaches can reveal phenotypes that are masked in loss-of-function mutants due to functional redundancy [170]. Major improvements to the CRISPRa system for its application in both monocots and dicots were made by Pan and colleagues using the CRISPR-Act3.0 system. They established dAaCas12b and SpRY dCas9 as more potent and widely applicable nucleases than the conventional dSpCas9 nuclease and enabled multiplexed gene activation of seven metabolic rice genes using a tRNA-guide RNA 2.0 expression system [182]. Moreover, by employing a catalytically active Cas9 in the CRISPR-Act3.0 system, simultaneous multiplexed genome editing and gene activation has been obtained [183]. This CRISPR-Combo approach has enormous potential for improving crop breeding, as demonstrated by its use for Arabidopsis speed breeding, accelerated regeneration and propagation of genome-edited *Populus* sp. (poplar) plants, and hormone-free regeneration of genome-edited rice [183]. Although tissue-specific CRISPR activation, stronger transcriptional activators and improved delivery of CRISPR/Cas machinery should broaden the use of CRISPRa in plants, the combination with genetic mapping and omics approaches already holds great potential for the combined identification and validation of immunity genes [170]. This idea is strengthened by the recent surge in CRISPRa applications for upregulating resistance-related plant genes and, consequently, the observation of improved disease resistance across multiple crops [170]. Additionally, due to the absence of actual genome editing and the reduced risk for off-target effects, application of CRISPRa and CRISPRi to differentially regulate targets for resistance breeding may face fewer regulatory hurdles [64,170].

### 3.2. Validation of NLR-Effector Combinations with Cellular Assays Based on Hypersensitivity Response

Recognition of a pathogen effector by its corresponding NLR protein triggers the hypersensitive response (HR), a rapid cell death that restricts pathogen spread. This HR-induced cell death serves as a readout for NLR-effector protein-mediated immunity and is widely used in effectoromics screening and NLR-effector pair validation. Typically, individual effectors are transiently expressed via agrobacterium infiltration in transgenic plants carrying the NLR genes, and those inducing HR are identified [184,185]. However, the Agrobacterium-mediated method has several limitations, including non-HR-related cell death, difficulties in co-expressing multiple candidates, and incompatibility with certain species, particularly monocots [186,187,188].

**Figure 5:**
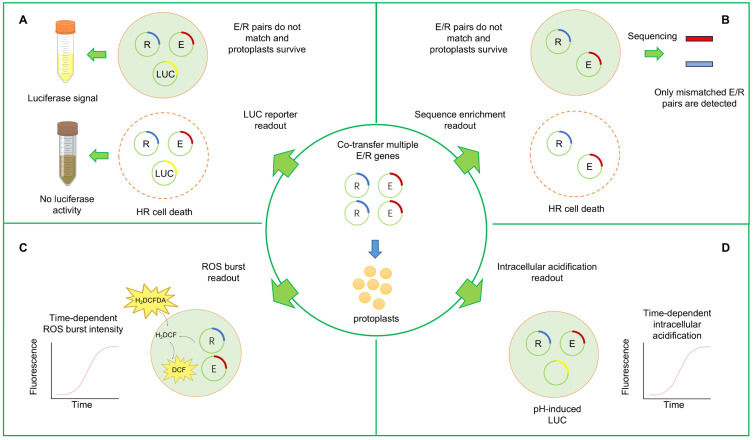
Methods for the detection of cell death triggered by the hypersensitive response or for generally monitoring plant immune responses in protoplasts that are co-transformed with NLR genes and effectors: (**A**) In co-transformed protoplasts where luciferase quenches or decreases, NLR proteins are involved in effector immunity. (**B**) Protoplasts that express a matching NLR-effector pair undergo cell death, and their corresponding NLR and effector readouts in RNA-sequencing decrease. (**C**) The ROS burst during the immune response can be detected through fluorescence from DCF in protoplasts. (**D**) Intracellular acidification during the immune response in can be monitored using a pH-induced luciferase reporter in protoplasts. R, NLR genes. E, effector. LUC, luciferase. Created in BioRender. Liu, Z. (2025) https://BioRender.com/xy82i4a (accessed on 20 January 2026).

Recently, the high-throughput detection of HR cell death in protoplasts has been employed to identify matching NLR-effector pairs (Figure 5A,B). Advances have occurred mainly in two aspects: (1) increasing effector or NLR gene expression throughput from one-to-one to pool-to-one assays; and (2) improving HR detection from fluorescence quenching caused by cell death to sequencing-based detection. This technique is primarily used to screen effector pools against a known NLR gene, but also holds promise for NLR gene screening.

For the throughput development, Saur et al. started by co-transfecting wheat protoplasts with a candidate NLR gene, effector, and luciferase reporter, where effective NLR-effector pairing triggered reduced luminescence [186]. As one-to-one screening was inefficient [186,189,190], Wilson et al. introduced a two-step pooling strategy based on initial screening with 25 effectors followed by sub-pools to identify causal effectors. They also developed a self-replicating plasmid, pWDV1, reducing the requirement for large amounts of high-purity DNA transfection [187]. However, pooled screening can cause insufficient expression of some effectors or NLRs, leading to false negatives. Arndell et al. recently optimized high-throughput screening by controlling the multiplicity of transfection, which represents the number of plasmid copies per cell. Adjusting the effector multiplicity of transfection to 0.07–0.7 million molecules per cell ensured each cell received only one or two constructs, enabling simultaneous delivery of up to ~700 effectors without major co-expression interference. Automated liquid handling was further used for uniform plasmid input and precision mixing [188].

For the HR cell death detection, early methods relied on fluorescence quenching to indicate cell death [186], but this was not always HR-specific. To improve specificity, Wilson et al. used the immunity gene promoter D14 to drive luciferase expression only upon NLR-effector recognition [187]. Alternatively, Arndell et al. extracted mRNA from surviving protoplasts and performed library-specific RNA-seq as a readout. Effectors triggering HR showed reduced transcript abundance in live cells, allowing identification via DESeq2 [188,191]. However, as cell death may not specifically be triggered by the hypersensitive response, monitoring dynamic immune responses via reactive oxygen species (ROS) bursts or intracellular acidification may serve as alternative readouts. These approaches use a dichlorofluorescin (DCF)-based system to monitor ROS production or a pH-induced luciferase reporter to monitor intracellular acidification during the immune response in protoplasts [192,193] (Figure 5C,D).

Despite these advances, the system has inherent limitations. Protoplasts lack cell walls, and NLR-effector interactions occur intracellularly, bypassing PRRs and the extracellular matrix in immunity. Relying solely on HR may also be incomplete: PEG-mediated transfection causes background cell death [194], and many immune responses, such as those involving *S* genes, do not trigger HR cell death. Furthermore, HR in protoplasts is not always equivalent to field-level resistance. Currently, HR assays can serve as screening tools, but final functional validation such as CRISPR is still needed.

On the other hand, while technically feasible, screening for unknown NLR genes using effector libraries could be challenging, mainly because there are fewer known effectors than known NLR genes [187]. Identifying relevant effectors is difficult, as bacterial and fungal pathogens may encode hundreds to thousands of uncharacterized ones. However, bacterial flagellin peptides have been employed in protoplast systems to screen receptor-like protein kinases [195], thereby substantiating the feasibility of this methodology.

## 4. Conclusion and Future Perspectives

### 4.1. Considerations for Method Selection

Recent advances in plant biotechnology, such as spatially resolved omics, proximity labeling, pooled CRISPR screens, and cell death assays, have expanded the repertoire of methods to identify and validate immunity genes. These methods, which rely on functional rather than genetic association to disease resistance, can reveal candidate genes previously hidden due to limited natural genetic variation. Additionally, they generate essential mechanistic insights, are more feasible for lab settings, and exploit the increasing applicability of CRISPR-induced genetic variation in resistance breeding. In this way, by increasing the relevance of candidate immunity gene lists and improving the subsequent validation capacity, these developments stimulate the ongoing transition from resistance breeding with broad QTLs to breeding with functional variants. However, this expanding methodological landscape to discover immunity genes also presents a challenge: selecting the most suitable approach for each situation requires careful consideration of multiple factors. Here, we provide preliminary selection guidelines based on research objectives, crop type, and experimental conditions:

(1) Selection based on research objectives.

For the identification resistance genes belonging to the NLR family, RenSeq-derived approaches such as MutRenSeq and AgRenSeq are highly effective. MutRenSeq is especially suitable when a clear resistant parental line is available and EMS mutagenesis is feasible, enabling direct cloning of causal NLR genes without extensive fine mapping. AgRenSeq is preferable when diverse germplasm panels are accessible and quantitative resistance needs to be resolved within the NLRome. When those methods generate a large candidate NLR genes list, high-throughput screening in protoplasts using HR cell death assays can help narrow down the candidates. Although it is not as conclusive as CRISPR knockout results, it offers a fast and practical way to prioritize the most promising genes for further validation.

When the objective is to uncover unbiased sources of genetic resistance especially those quantitative or minor-effect resistance loci, approaches dependent of strong genetic association become particularly valuable. GWAS combined with multi-omics can prioritize candidate genes within large QTL intervals. Alternatively, single-cell RNA sequencing and spatial omics are well-suited for identifying cell-type-specific regulators that may not be detectable through bulk tissue analysis. These strategies are particularly advantageous for dissecting complex resistance mechanisms.

For mechanistic dissection of plant-pathogen interactions, interactomics-based approaches such as proximity labeling, RNA-protein complex isolation, and computational protein–protein interaction prediction are recommended. These methods provide direct molecular evidence and can reveal susceptibility factors or immune hubs that may not show strong genetic signals.

(2) Selection based on crop characteristics

For crops with large and complex genomes (e.g., wheat or potato), RenSeq-derived approaches are particularly advantageous due to their ability to reduce genome complexity and focus on NLR genes. RenSeq-derived methods are also suitable for less frequently studied crops and wild relative species without reliable genome data, as NLR probes can be designed based on related species. In contrast, for species with well-annotated genomes, multi-omics approaches enable unbiased exploration of a wide range of immunity-related genes.

For biotechnology-friendly model plants, such as tomato and Arabidopsis, efficient protoplast isolation makes them well-suited for single-cell omics, especially when different cell types exhibit distinct susceptibility phenotypes. Cell death assays are also based on protoplast systems, but they require optimized high-throughput transformation techniques. In addition, a well-established plant transformation system is a prerequisite for conducting CRISPR screens. Interactomics, such as the study of protein–protein interaction, is suitable for well-known pathogens and plants, while RNA-protein interactions are gaining increasing attention in the field of RNA virus research.

In species with low natural genetic diversity limited germplasm resources, function-based approaches, such as scRNA-seq and proximity labeling, can compensate for the limitations of traditional mapping. Conversely, when crops possess rich genetic diversity, clear population structure, and extensive phenotypic variation within natural populations, GWAS can uncover valuable disease resistance alleles from these variations. Integrating GWAS with multi-omics data further narrows candidate genes within QTL regions.

(3) Selection based on laboratory conditions

Large-scale genetic experiments, including GWAS and QTL mapping, require extensive field or greenhouse facilities to support large populations. Similarly, MutRenSeq and AgRenSeq often demand substantial experimental space, as they involve EMS mutagenesis or the maintenance of diverse germplasm panels. In contrast, biochemistry-based interaction assays generally have lower space requirements and can be performed under more controlled laboratory conditions. Large-scale CRISPR screens also depend on sufficient facilities to support high-throughput plant transformation and regeneration. However, HR cell death assays based on protoplast systems can significantly reduce space requirements, as they bypass the need for whole-plant cultivation.

Omics-based studies require access to specialized platforms, such as high-throughput sequencing and mass spectrometry. The integration of multiple omics layers can further increase the power but also the costs substantially. Although computational approaches reduce certain experimental demands, they depend strongly on computational infrastructure and processing power.

Overall, the choice of methods should be driven by clear scientific logic rooted in the research objectives and goals. At the same time, it requires a comprehensive evaluation of experimental feasibility, technical practicality, and realistic expectations regarding time and cost.

### 4.2. Limitations and Future Directions in Resistance Breeding

Despite substantial methodological advances, several limitations continue to hamper progress in plant resistance breeding. The immune system of plants is highly complex, and the discovery of canonical resistance genes such as NLRs and PRRs remains insufficient [39]. Meanwhile, numerous unbiased genomic studies have identified resistance mechanisms distinct from these canonical genes [17], indicating that non-NLR and quantitative resistance mechanisms are also underexplored. The polygenic and environment-dependent nature of durable resistance complicates genetic dissection, often leading to unstable association signals in traditional genetic studies [62]. Emerging function-based methods show clear advantages in dissecting disease resistance mechanisms, accelerating gene identification, and reducing reliance on natural variation resources. However, the application of these approaches depends on increasingly complex experimental techniques that all have their own limitations. For instance, the efficient isolation of RNA-protein complexes in plants [161] and high-throughput protoplast transformation still require technical breakthroughs [188]. The optimization of computational algorithms, particularly the application of machine learning in plant disease resistance research, relies largely on early models such as random forests and support vector machines. In addition, functional validation relies on biochemical assays and transformation validation, which lag far behind the pace of candidate gene discovery. To fully exploit the emerging methods for candidate immunity gene discovery, approaches that facilitate functional validation of all candidate genes is essential. High-throughput gene functional validation platforms, such as CRISPR screening, are therefore in urgent need of optimization and upscaling.

Given these challenges, solutions lie not only in continuous pursuit of technological breakthroughs, but also in the promising future direction of synergistically integrating multiple approaches. For example, incorporating transcriptomic, metabolomic, or epigenomic data into GWAS frameworks can substantially refine candidate gene prioritization within large QTL intervals [23,24,25]. Multi-omics integration is particularly powerful for linking resistance-associated metabolites to genes within their biosynthetic pathways, potentially uncovering the causal variants underlying complex resistance traits. Likewise, high-throughput computational prediction of protein–protein interaction can complement experimental interactomics by prioritizing biologically meaningful interactions for downstream validation [36].

However, realizing this integrative potential requires robust, user-friendly analytical platforms capable of handling complex, multi-dimensional datasets. Standardized pipelines for data integration, improved phenotyping accuracy, scalable gene-editing systems, and accessible computational infrastructure will be essential. Ultimately, the future of resistance breeding depends not only on discovering new resistance genes, but on strategically combining complementary technologies into a coherent and translational pipeline that bridges gene discovery, functional validation, and durable deployment.

## Figures and Tables

**Table 1:** Comparison of the key characteristics of emerging methods to identify and validate plant immunity genes.

Category	Methods		Principle	Main Advantages	Main Limitations	Target Genes
**Genetic methods**	Map-based cloning		Linkage or association between phenotype and genomic region	• Unbiased approach that scans the entire genome independent of gene function, allowing for the discovery of completely novel loci • Direct link between phenotype and candidate genes • Exploits genetic diversity available in natural association panels	• Requires large-scale phenotyping, extensive fine mapping, and substantial time and resources → less accessible for labs • Difficult to detect minor effect and rare alleles, structural variants and non-sequence-based variations • Resolution of genetic mapping depends on the genetic diversity, size and structure of the population	All types, with bias for qualitative or major quantitative effect loci
	QTL mapping					
	GWAS					
**Emerging methods alternative to genetics**	**Targeting NLR genes**	NLR gene annotation	Annotation of NLR genes through conserved sequences	• High-throughput, rapid and cost-effective • Correct NLR annotation is beneficial for all candidate gene lists • Complementarity with RenSeq-derived methods	• Predicted NLRs lack direct pathogen/effector association • Dependent on high-quality sequence data • Advances require more functionally characterized NLRs	NLR genes
		MutRenSeq andAgRenSeq	Sequence enrichment and alignment of NLR genes	• Requires less phenotyping and sequencing than unbiased mapping •Directly associates NLRs with resistance, without fine mapping • Suitable for crops with complex genomes (e.g., wheat and potato)	• Limited to NLR gene family, while other types of immunity genes could confer actual resistance (e.g., S or PRR genes) •Still depends on accurate phenotyping	NLR genes
	**Omics**	Transcriptomics Proteomics Metabolomics	Comparative profiling of biomolecules (RNA, proteins and metabolites) mediating resistance	• Independent of natural genetic variation • Can identify all immunity gene types and minor quantitative loci • Smaller scale and rapid experimental timeline → more accessible • Spatial resolution, e.g., scRNA-seq and spatial proteomics, with demonstrated use cases for plant-pathogen research	• Differential regulation = relatively weak functional correlation to resistance → very dependent on validation • Methodological advances, such as spatially resolved omics, are technically challenging for plant tissues • Variable length of candidate gene lists (depends on setup)	Unbiased (all types)
	**Interactomics**	Proximity labeling	Biotinylation and isolation of proteins adjacent to bait protein of interest	• Independent of natural genetic variation • Detects all types of native interactions in planta, including weak and transient interactions • Applicable to specific subcellular structures	• Risk of substrate cytotoxicity • Large enzyme size may affect protein localization and function, potentially leading to false positive results • Background from endogenous biotinylated plant proteins	Interactors with pathogen, bias for susceptibility genes
		Protein–protein interaction prediction	Computational PPI prediction based on sequence and structure	• High-throughput, rapid and cost-effective • Accelerates experimental interactomics research • Benefit from improved machine learning models	• Accuracy depends on experimentally validated PPI data • Predicted PPIs always require experimental validation → need for high-throughput interaction validation platforms • Computationally intensive	Interactors with pathogen, no bias
		Viral RNA-protein complex isolation	Isolation and identification of proteins bound to viral RNA	• Elucidates RNA-protein interactions, which are essential to infect hosts but are missed by protein–protein interaction studies • Crosslinking enables detection of weak or transient interactions	• Technically challenging, particularly in plant systems • Specific to plant RNA viruses • Most examples are from human RNA virus research	Interactors with pathogen, bias is unknown
**Emerging methods for validation**	CRISPR screening		High-throughput generation of mutant knockout lines	• Transformation of pooled gRNA libraries enables higher-throughput candidate gene validation • Enables validation independent of available genetic variation • Increasing applicability of gene editing in resistance breeding • Promising alternatives for silencing/overexpression: CRISPRi/a	• Remains labor- and time-consuming to generate and analyze large mutant populations for resistance • Pleiotropic effects or lethality could hide beneficial phenotype of candidate gene knockouts • Risk of off-target editing effects	Unbiased
	Hypersensitive response cell death assay		Hypersensitive response cell death triggered by effector-NLR gene pairs	• Avoids the need for stable transformation • Directly validates immunity reaction in protoplasts • Knowledge of specific effector recognized by NLR genes = valuable information for follow-up research	• NLR gene-specific → not applicable for PRRs, *S* genes, … • Application for NLR instead of effector screening has not been described yet • Challenging without prior knowledge on specific effectors • Risk of false positive cell death phenotype unrelated to HR	NLR genes

## Data Availability

Data availability is not applicable to this article as no new data were created or analyzed in this study.
